# IL-1b in the Secretomes of MSCs Seeded on Human Decellularized Allogeneic Bone Promotes Angiogenesis

**DOI:** 10.3390/ijms232315301

**Published:** 2022-12-04

**Authors:** Yi-Chun Chen, Yu-Show Fu, Shang-Wen Tsai, Po-Kuei Wu, Chao-Ming Chen, Wei-Ming Chen, Cheng-Fong Chen

**Affiliations:** 1 Department of Orthopaedics and Traumatology, Taipei Veterans General Hospital, Taipei 11217, Taiwan; 2 Department of Anatomy and Cell Biology, Faculty of Medicine, National Yang Ming Chiao Tung University, Taipei 11221, Taiwan; 3 Department of Orthopaedics, School of Medicine, National Yang-Ming Chiao Tung University, Taipei 11221, Taiwan

**Keywords:** secretome, MSC, allogeneic bone, angiogenesis, cytokine

## Abstract

Angiogenesis plays an important role in the development of bone and bone regeneration to provide the required molecules. Mesenchymal stem cells (MSCs) are pluripotent, self-renewing, and spindle-shaped cells, which can differentiate into multiple lineages such as chondrocytes, osteocytes, and adipocytes. MSCs derived from bone marrow (BMMSCs), adipose tissue (ADMSCs), and Wharton’s jelly (UCMSCs) are popular in the field of tissue regeneration. MSCs have been proposed that can promote bone regeneration by enhancing vascularization. In this study, the angiogenic potential of secretomes of undifferentiated and osteo-differentiated BMMSCs, ADMSCs, and UCMSCs seeded on human decellularized allogeneic bone were compared. Human umbilical vein endothelial cells (HUVECs) were treated with MSC secretomes. Cell growth, cell migration, and angiogenesis of HUVECs were analyzed by MTT, wound healing, and tube formation assays. Angiogenic gene expression levels of MSCs were evaluated using real-time quantitative PCR. Antibody neutralization was performed to validate the candidate target. Our study demonstrates that the angiogenic gene expression profile is tissue-dependent and the angiogenic ability of secretomes is independent of the state of differentiation. We also explore that IL-1b is important for MSC angiogenic potential. Taken together, this study proves that IL-1b in the secretomes plays a vital role in angiogenesis.

## 1. Introduction

The formation of blood vessels is a critical process during bone development and regeneration. Bone is a highly vascularized tissue, and bone defects such as fractures usually contribute to the loss of nutrients, growth factors, calcium, and many other substances needed for cells to form a new bone. In bone fractures, the architecture of bone and blood vessels is disrupted at the site of injury. Consequently, the formation of fracture hematoma occurs, which contains immune and bone marrow cells, and this process is called the inflammation phase, an initiation of fracture healing [1,2].

During inflammation, the balanced expression of cytokines is critical to establishing a suitable microenvironment for angiogenesis and the regeneration of bone defects. Proinflammatory cytokines such as tumor necrosis factor alpha (TNFa) [3], interleukin (IL)-1 [4], and IL-6 [5] have been reported that can be pro-angiogenic factors.

Bone marrow, adipose tissue, and Wharton’s jelly are the major sources of MSCs. MSCs are adult stem cells with multiple differentiation capabilities and are regarded as potential cells for tissue repair [6,7,8]. They are immune privileged because the major histocompatibility (MHC) Class I antigens are normally expressed on their surface but not Class II. In addition, researchers have reported that MSCs can secret soluble factors to regulate immune responses [9,10,11]. Due to the variety of sources, the ability of immunomodulation, and multiple differentiation potential, MSCs are employed for plastic and orthopedic reconstruction [12,13]. For tissue regeneration, MSCs can modify the microenvironment to enhance angiogenesis in situ. MSCs create a suitable microenvironment by secreting proinflammatory, chemotactic, and angiogenic factors to recruit endothelial cells’ ingrowth [14]. For example, during bone formation, MSCs with biodegradable scaffolds are capable of promoting osteogenesis and angiogenesis in vivo [15].

Biodegradable scaffolds are promising materials for bone reconstruction. Inorganic biomaterials such as calcium phosphate-based scaffolds have been intensively studied [16,17,18]. Although synthetic biomaterials have great advances, their properties, for instance, porosity, surface, and mechanical strength, are needed to be further improved to recapitulate the bone microenvironment. Decellularized matrix has been employed in tissue engineering such as in skin, tendon, cartilage, and bone [19,20]. The main purpose of decellularization is to preserve the architecture and extracellular matrix of tissues. In our previous study, we demonstrated that the human decellularized bone (hDCB) matrix could be a scaffold for osteogenesis or chondrogenesis of MSCs because of its natural properties [21].

The cell culture microenvironment has been intensively studied, especially for comparing 2D culture conditions to 3D ones. Cells cultured on 3D scaffolds can secrete more bioactive molecules that promote cell growth, migration, and differentiation. For example, an electrospun fiber scaffold seeded with MSCs could produce a five times higher concentration of HGF and ICAM-1, promote wound healing, and reduce scar formation in the cornea wound healing model when compared to 2D cultures [22]. Uncovering the angiogenic potential of secretomes of undifferentiated and osteo-differentiated MSCs derived from different tissue origins is important for clinical practice. Due to the promising results of 3D scaffolds in regenerative medicine, we would like to further explore and compare the angiogenic potential of secretomes derived from undifferentiated and osteo-differentiated BMMSCs, ADMSCs, and UCMSCs cultured on the hDCBs. It is expected that this study will help to answer if the tissue of origin and differentiation state of MSCs have underlying differences in angiogenesis. The function of MSC secretomes on cell growth was assessed using MTT assays. The cell migration capability of MSC secretomes was analyzed by wound healing assays. The expression of angiogenic genes of MSCs was evaluated by real-time quantitative PCR. To analyze the angiogenic potential and evaluate the candidate target of MSC secretomes, we performed in vitro tube formation assays using human umbilical cord endothelial cells (HUVECs) and an antibody neutralization experiment.

## 2. Results

### 2.1. MSC-CMs Promote Cell Growth

**Figure 1:**
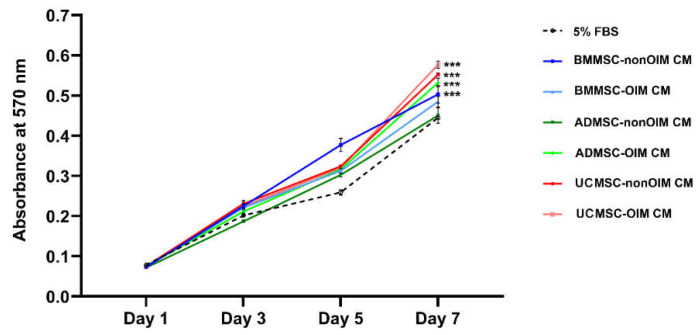
Cell proliferation of human umbilical vein endothelial cells treated with secretomes derived from osteo-induced (OIM) or noninduced (nonOIM) MSCs cultured on human decellularized bone matrix. The results showed that most of conditioned media could promote cell growth compared to the medium containing 5% FBS. Osteo-induced and noninduced UCMSC conditioned media had a higher potential to induce cell proliferation compared to the other groups. The absorbance at each timepoint represents mean ± SEM. A one-way ANOVA with Tukey post hoc test was used to determine statistical significance at day 7 (*** *p* < 0.001).

To explore the function of MSC-CMs in cell growth, MTT assays, colorimetric assays using 3-(4,5-dimethylthiazolyl-2)-2,5-diphenyltetrazolium bromide (MTT) which could be reduced by cellular enzymes to an insoluble purple product formazan, were performed (Figure 1). The growth rates of endothelial cells treated with MSC-CMs were recorded on days 1, 3, 5, and 7. The results showed that BMMSC- and ADMSC-nonOIM CM-treated groups had a smooth growth rate from day 1 to day 7. On the other hand, the other groups showed noticeable increases in growth rates from day 5 to day 7. In addition, the data showed that UCMSC-OIM CM had the highest potential (*p* < 0.001) to promote cell growth compared to the 5% FBS group on day 7. Other groups, UCMSC-nonOIM CM, ADMSC-OIM CM, and BMMSC-nonOIM CM, also showed significant differences (*p* < 0.001) compared to the 5% FBS group on day 7. Although BMMSC-OIM CM had a higher potential in promoting cell growth, it did not reach the statistical difference (*p* > 0.05) compared to 5% FBS on day 7.

### 2.2. The Effect of MSC-CMs on Cell Migration

**Figure 2:**
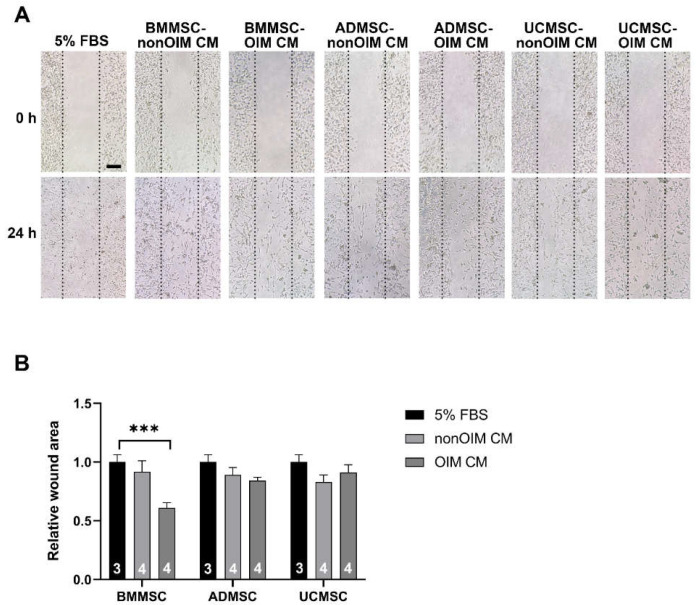
Cell migration was analyzed by wound healing assay using HUVEC cells. (**A**) Representative images showed the results of cell migration at 0 h and 24 h. (**B**) The wound closure of each group was normalized with the 5% FBS group and expressed as a relative wound area. Data were analyzed using a one-way ANOVA with Tukey’s post hoc test to determine statistical significance. Bar graphs present mean ± SEM. The number of replicates is inside the bars. The scale bar is 100 mm. Statistical significance is present as *** *p* < 0.001 (compared to 5% FBS) and the results do not reach the statistical differences that are not shown.

Cell migration is an essential process for angiogenesis. The cell migration was evaluated using HUVECs treated with 5% FBS (as a control) or MSC-CMs. As shown in Figure 2, the 5% FBS-treated group did promote cell migration as expected. In the MSC-CM-treated groups, the data showed that BMMSC-OIM CM had the lowest capacity to induce cell migration compared to 5% FBS (*p* < 0.001). Furthermore, the other groups did not show significant differences compared to the 5% FBS-treated group (*p* > 0.05).

### 2.3. Analysis of Angiogenic Potential of MSC Secretomes by Tube Formation Assay

**Figure 3:**
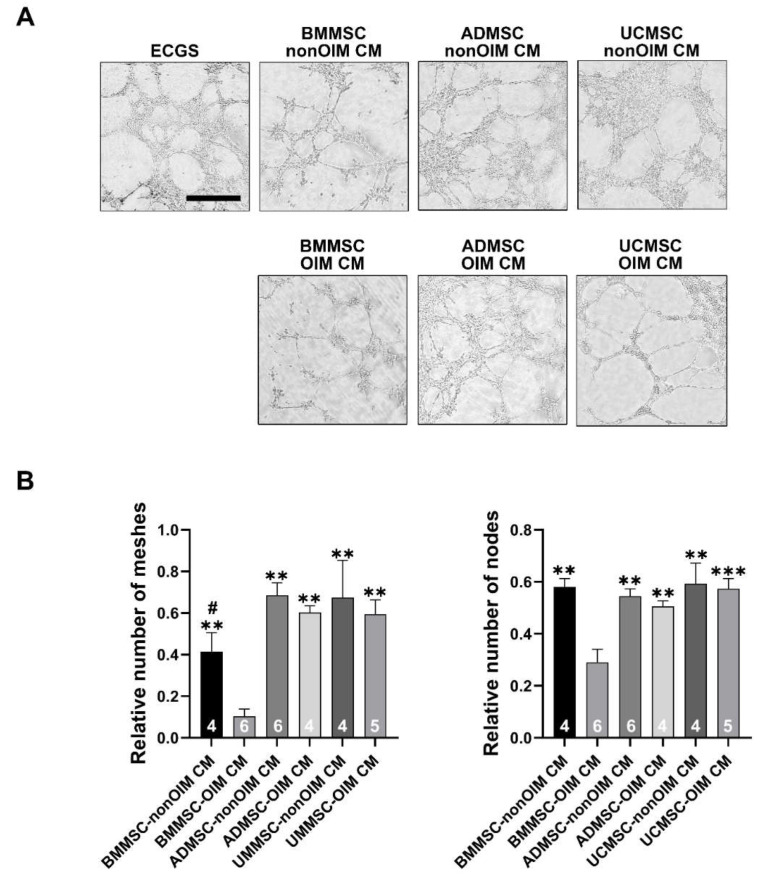
Tube formation assay for analyzing the angiogenic potential of MSC secretomes. (**A**) Micrographs of tube formation of HUVECs treated with osteo-induced (OIM) or noninduced (nonOIM) MSC-CMs. (**B**) CM groups were normalized with the ECGS group and expressed as a relative number of meshes and nodes. Bars are presented as mean ± SEM. The number of replicates is inside the bars. A one-way ANOVA with Tukey’s post hoc test was used to determine statistical significance (** *p* < 0.01, *** *p* < 0.001 vs. BMMSC-OIM CM; # *p* < 0.05 vs. ADMSC-nonOIM CM). Scale bars are 500 μm.

The angiogenic capacities of MSC secretomes were evaluated by in vitro tube formation assay using HUVEC cells. One microgram per milliliter of MSC secretome was added into serum-free media to assess the angiogenic potential, and 1% of ECGS (endothelial cell growth supplement) was a positive control. All the CM groups were normalized to the ECGS group for statistical analyses. The relative numbers of meshes and nodes were readouts to evaluate the angiogenic capacities. The addition of MSC secretomes into serum-free media did induce HUVECs to form vascular structures. The nonsignificant differences could be investigated between 5% FBS and CM-treated groups, although the BMMSC-OIM CM group showed the lowest angiogenic potential compared to other groups (*p* < 0.05 compared to 5% FBS) (Figure 3A,B).

### 2.4. qPCR Profiling of Angiogenic Genes

**Figure 4:**
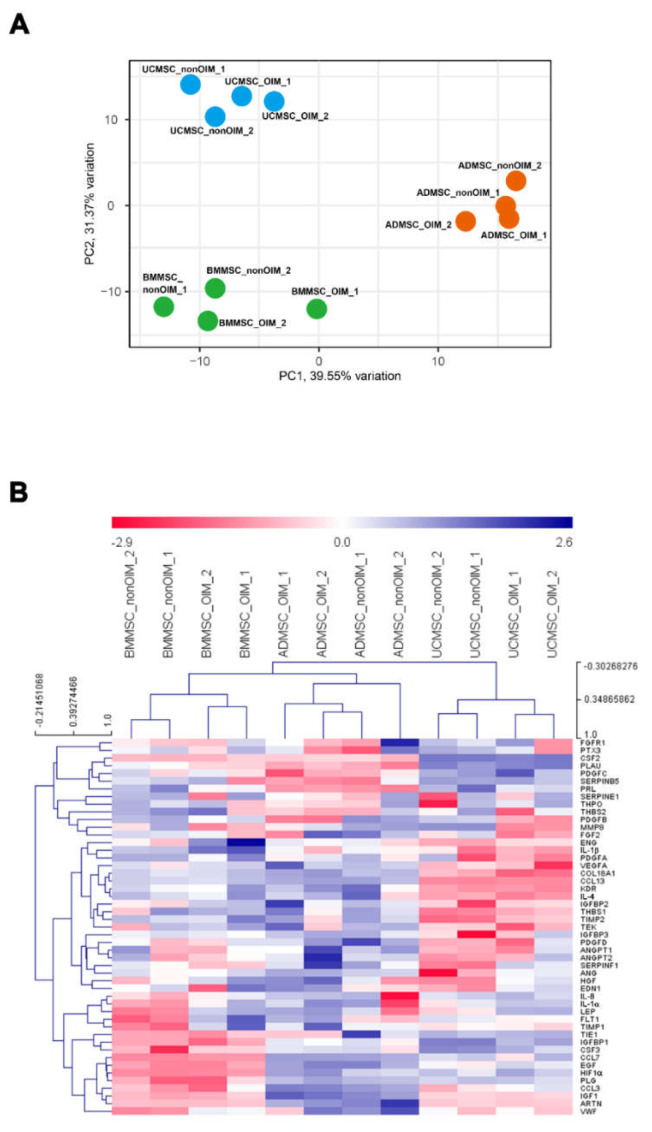
Data visualization of DCt values after qPCR. (**A**) A principal component analysis (PCA) of DCt values. The PCA plot was from the viewpoint of the samples, and variances of PC1 and PC2 were 39.55% and 31.37%, respectively. (**B**) A hierarchical clustering (HCL) of genes and samples. Red and blue colors represented higher and lower expression levels of genes, respectively. DCt values were normalized using *GAPDH* as a reference gene.

The angiogenic gene panel was selected and examined using the qPCR technique. The gene expression profiles of MSCs derived from different tissue origins were classified by principal component analysis (PCA). The result showed that BMMSCs, ADMSCs, and UCMSCs were separated into three groups and were independent of osteogenic differentiation (Figure 4A). The data were further visualized using the multiple experiment viewer (MeV) software, and the hierarchical clustering was performed based on samples and genes (Figure 4B). The hierarchical clustering showed the same result as PCA analysis, which showed that BMMSCs, ADMSCs, and UCMSCs were also separated into three groups and showed different gene expression patterns.

**Figure 5:**
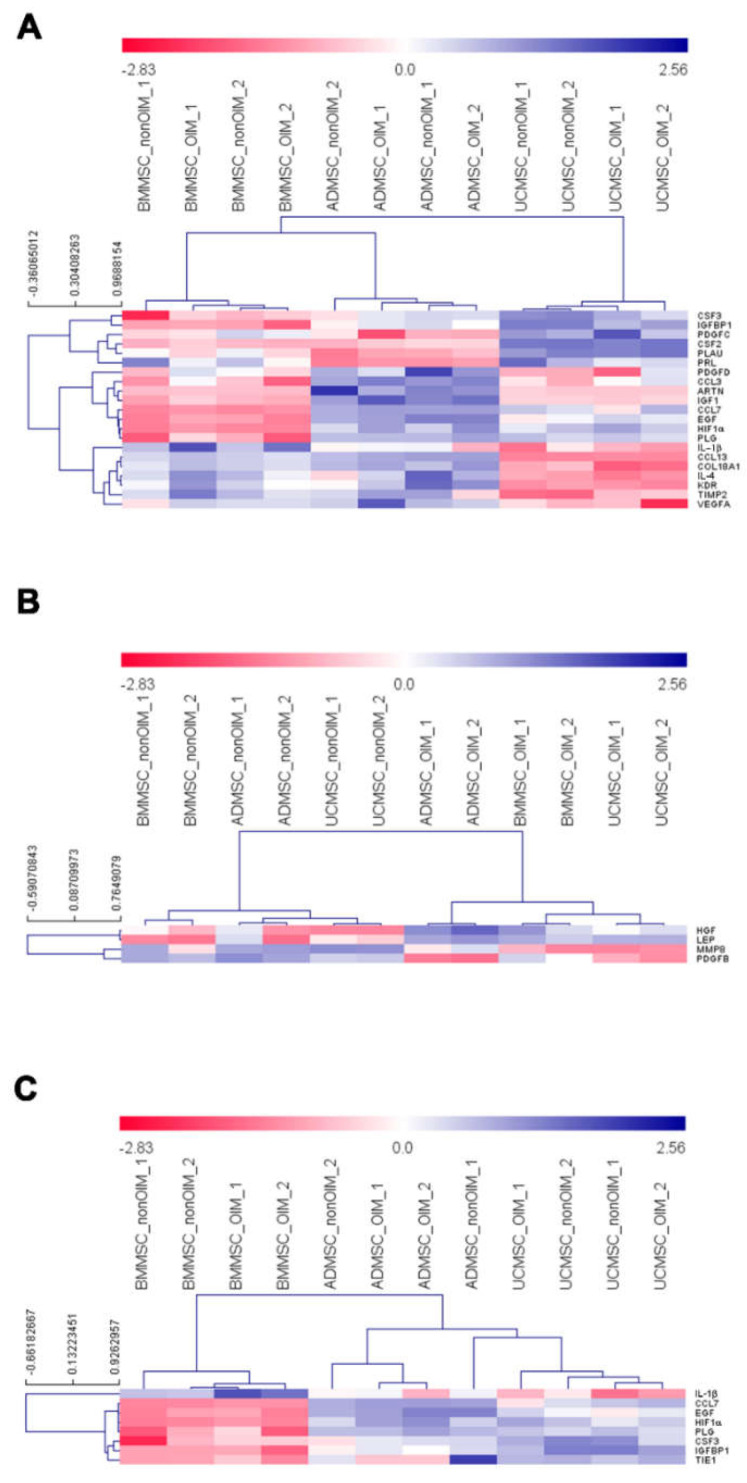
Differentially expressed gene analyses. (**A**) A one-way ANOVA analysis showed the differentially expressed genes among MSCs from different sources. Permutations (1000), Pearson correlation, HCL clustering with average linkage, and *p*-value < 0.01 were used. (**B**) A *t*-test examined the differentially expressed genes between nonOIM and OIM samples. A *p*-value < 0.01 was used. (**C**) A *t*-test explored the differentially expressed genes between BMMSCs and the other two MSCs. A *p*-value < 0.01 was used. Red and blue colors represent high and low gene expression levels, respectively.

We used the MeV software to analyze the expression profiles of differentially expressed genes. First, a one-way ANOVA was employed to analyze the differentially expressed genes among BMMSCs, ADMSCs, and UCMSCs. The results showed the different expression profiles of angiogenic genes in BMMSCs, ADMSCs, and UCMSCs. For example, the low expression levels of *CCL3*, *ARTN*, and *IGF1* were in BMMSCs and UCMSCs but were high in ADMSCs (Figure 5A). Second, a *t*-test was utilized to perform the statistical analysis to uncover the differentially expressed genes between nonOIM and OIM groups. The result showed a few differentially expressed genes, such as that the expression level of *HGF* and *LEP* in the nonOIM group was higher than that of the OIM group and the expression level of *MMP8* and *PDGFB* in the nonOIM group was lower than that of the OIM group (Figure 5B). Finally, the differentially expressed genes were further analyzed by comparing BMMSCs to the other two MSCs. The data showed that the expression levels of *CCL7*, *EGF*, *HIF1a*, *PLG*, *CSF3*, *IGFBP1*, and *TIE1* in BMMSCs were lower than ADMSCs and UCMSCs. *IL-1b* was high in BMMSCs but low in ADMSCs and UCMSCs (Figure 5C).

### 2.5. Validation of Analyzed Results by IL-1b Neutralization

**Figure 6:**
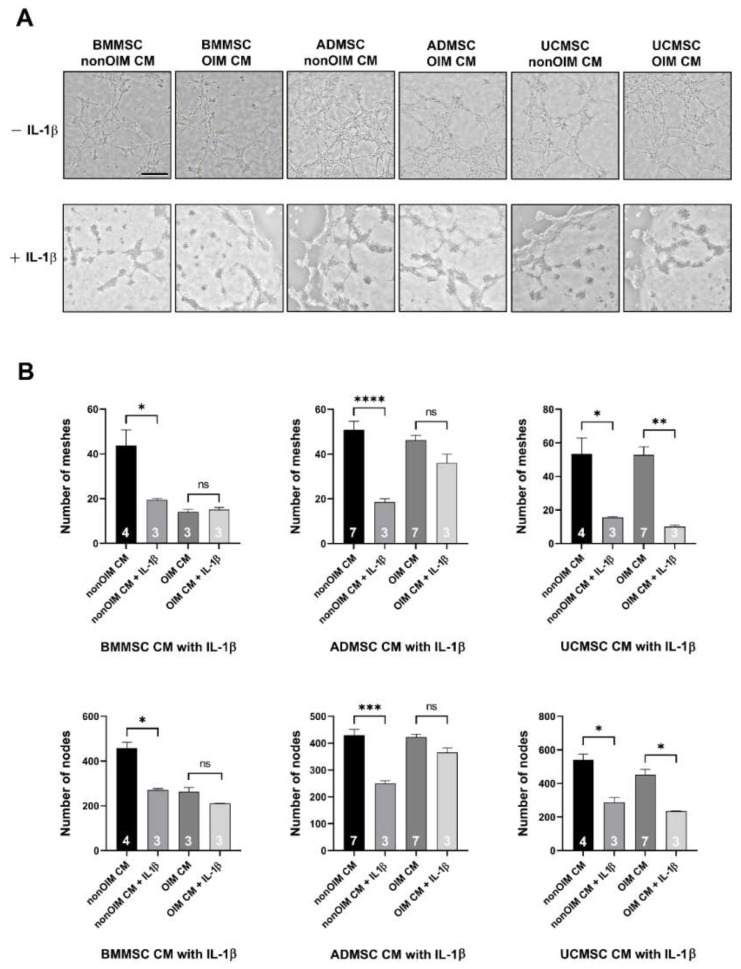
IL-1b neutralization compromised the angiogenic capability of MSC-CMs. (**A**) Phase contrast images of in vitro tube formation assay using HUVECs treated with MSC-CMs with or without IL-1b antibody. (**B**) The statistical results of the number of meshes and nodes were to evaluate the angiogenic potential of MSC-CMs with or without IL-1b antibody. The number of replicates is inside the bars. The scale bar is 500 mm; ns represent not significant; * *p* < 0.05 ** *p* < 0.01, *** *p* < 0.001 and **** *p* < 0.0001.

*IL-1b* was selected to validate the analyzed results because it was the only gene with a higher expression level in BMMSCs compared to ADMSCs and UCMSCs. The results revealed that BMMSC, ADMSC, and UCMSC-CMs neutralized with the human recombinant IL-1b antibody could lead to the compromised angiogenesis of HUVECs (Figure 6A). The numbers of meshes and nodes were further analyzed by ImageJ software with the angiogenesis analyzer plugin (Figure 6B). The IL-1b neutralized BMMSC-nonOIM CM showed significant decreases (*p* < 0.05) in the numbers of meshes and nodes compared to the non-neutralized group. However, there was no difference observed in the IL-1b neutralized BMMSC-OIM CM group and the non-neutralized group (*p* > 0.05). In the ADMSC-nonOIM CM groups, the addition of the IL-1b antibody led to significantly decreased numbers of meshes and nodes (*p* < 0.001). In the IL-1b neutralized and non-neutralized ADMSC-OIM CM groups, there were no significant differences in the numbers of meshes and nodes (*p* > 0.05). The neutralization of IL-1b led to significantly reduced numbers of meshes and nodes not only in the UCMSC-nonOIM CM group but also in the UCMSC-OIM CM group (*p* < 0.05). Taken together, IL-1b in the secretomes of MSCs plays a vital role in regulating angiogenesis.

## 3. Discussion

The development of regenerative medicine has made remarkable progress in recent years. Although regenerative medicine of cell-based study is widely evaluated, its effectiveness depends on the interaction between cells and the surrounding microenvironment [23,24,25]. Regarding MSCs, they can derive from different tissue origins and possess differential bioactivity in tissue regeneration, especially for angiogenesis [26,27,28]. Thus, the mesenchymal stem cell niche is important to recapitulate the tissue of origins [22]. In this study, we seeded MSCs on human decellularized allogeneic bone to provide a suitable microenvironment for producing more bioactive molecules. First, the functions of MSC secretomes on cell growth and migration were evaluated. The results demonstrated that most MSC secretomes could promote cell proliferation and migration analyzed by MTT and wound healing assays, respectively. Moreover, the effects of MSC secretomes on angiogenesis were also demonstrated. To unveil the potential genes involved in angiogenesis, real-time quantitative PCR was employed. The results showed that the expression pattern of selected genes was independent of the state of differentiation. According to the PCA and heatmap analyses, samples were grouped by their tissue origins. The differentially expressed genes showed a unique pattern based on the tissue origins of MSCs, such as that the low expression levels of *CCL3*, *ARTN*, and *IGF1* were in BMMSCs and UCMSCs but were high in ADMSCs. Advanced analysis of differentially expressed genes according to noninduced (nonOIM) and osteo-induced (OIM) groups revealed that a few genes were clustered, such as the higher expression levels of *HGF* and *LEP* in the nonOIM group than that of the OIM group and the lower expression levels of *MMP8* and *PDGFB* in the nonOIM than that of the OIM group. Finally, the differentially expressed genes in ADMSCs and UCMSCs compared to BMMSCs were further analyzed. We found that *IL-1b* was a unique gene and the expression level was low in BMMSCs compared to ADMSCs and UCMSCs. The validation was conducted using in vitro tube formation. The results showed that IL-1b does possess the pro-angiogenic capacity in the MSC secretomes. Most of the vascular networks were compromised after IL-1 b neutralization.

Hoch et al. proposed that the differentiation state of MSCs could affect their angiogenic potential [29]. MSCs cultured in growth media (GM, non-induction) expressed a higher level of *VEGFA* compared to OM (osteogenic media without dexamethasone) and OM+ (osteogenic media with 10 nM dexamethasone). In addition, GM-cultured MSCs could produce higher levels of angiogenic cytokines such as IL-6, IL-8, and MCP-1. In our study, we found that the secretomes of nonOIM MSCs had a higher angiogenic potential compared to OIM MSCs, but the results did not reach statistical significance. Furthermore, we compared the expression profiles of angiogenic genes using real-time quantitative PCR. Although the gene expression patterns were independent of the state of osteogenic differentiation, the gene expression patterns were highly correlated to the tissue origins of MSCs. The controversial results between our study and that of Hoch et al. might be due to the culture environment. We grew MSCs on hDCB scaffolds, but Hoch et al. cultured cells in the dishes.

It has long been known that inflammation can trigger angiogenesis through the cytokines secreted from macrophages, neutrophils, lymphocytes, etc. [30]. IL-1b, a proinflammatory cytokine, was proposed as a potential angiogenic factor in Lewis lung carcinoma [31]. Inflammatory cytokines secreted from bone marrow mesenchymal stem cells derived from proximal humerus fracture have been demonstrated, although the protein expression level of IL-1b is low [32]. In ADMSCs it has been shown that the secretion of bioactive factors such as chemotactic protein 1 (MCP-1), colony stimulator of granulocyte-macrophage factor (GM-CSF), hepatocyte growth factor (HGF), interleukin-6 (IL-6), and IL-8 is better than BMMSCs [33]. Wharton’s jelly, a gelatinous connective tissue within the umbilical cord, is another source of MSC. When comparing Wharton’s jelly MSCs to BMMSCs and ADMSCs in the secretion of cytokine, Wharton’s jelly MSCs secrete higher amounts of proinflammatory cytokines but BMMSCs and ADMSCs present a higher angiogenic profile [34]. A previous study uncovered the protein expression profiles of secretomes derived from 2D-cultured BMMSCs, ADMSCs, and UCMSCs using LC-MS/MS, and the angiogenic capacities are also evaluated [35]. They proposed that Wharton’s jelly MSCs have the highest potency in inflammation-induced angiogenesis compared to BMMSCs and ADMSCs. Targeting a specific gene or protein to confirm the analyzed results was missing, although they identified hundreds to thousands of proteins differentially expressed in these MSC-CMs. Generally, our data showed that the secretomes of UCMSCs and ADMSCs could enhance cell proliferation compared to BMMSCs. In cell migration and angiogenesis, most MSC secretomes possessed equivalent bioactivities, but the secretome of BMMSC OIM showed the lowest potency. Regarding the impact of the microenvironment on cellular function and biological activity, culturing MSCs on a three-dimensional microenvironment can provide a favorable niche and improve therapeutic outcomes, such as the application of the MSC secretome in corneal wound healing [22]. A recent study proposed that the three-dimensional culture of MSCs can enhance the production of cytokines which may have therapeutic benefits [36].

Gene expression can be measured at RNA or protein levels. Real-time quantitative PCR is a powerful and sensitive technique for detecting a gene expression level from RNA and can be an alternative reading to protein expression [37]. In this study, we employed real-time quantitative PCR to evaluate the selected angiogenic gene expression profiles of MSCs and performed the functional analysis to confirm one of our findings. In antibody neutralization experiments, we utilized the IL-1b antibody to inhibit the angiogenic ability of MSC secretomes. The results showed that the IL-1b antibody could constrain the angiogenic capability in most MSC secretomes, with exceptions in BMMSC and ADMSC OIM secretomes. The limited inhibitory effect might be due to the poor angiogenic ability of the BMMSC OIM secretome, so we cannot investigate a further depression of angiogenesis after IL-1b neutralization. In the ADMSC OIM secretome, we considered that the amount of IL-1b antibody might be not enough to suppress the angiogenic ability of IL-1b in the ADMSC OIM CM. In future studies, we need to learn more about the angiogenic potential of MSCs and their secretomes in our culture environment using advanced techniques such as RNA-seq or proteomics to explore more genes or proteins. There are some limitations in this study: (1) the real interaction between MSCs and the in vivo microenvironment cannot be fully reproduced, although the application of human decellularized allogenic bone in vitro; (2) the dual role of IL-1b in inflammation and angiogenesis should be further studied by a feasible in vitro or in vivo model; (3) the angiogenic potential of MSC secretomes from our culture system has to be validated via an animal study, such as in mice.

## 4. Materials and Methods

### 4.1. MSC Culture

Human BMMSCs, ADMSCs, and UCMSCs were cultured according to our previous study [21]. Briefly, MSCs were seeded in 10 cm culture dishes and cultured in an α-modified Eagle’s medium (α-MEM; Thermo Fisher Scientific, Waltham, MA, USA) supplemented with 10% fetal bovine serum (FBS; Thermo Fisher Scientific, Waltham, MA, USA), penicillin (100 μg/mL), and streptomycin (100 μg/mL) and incubated at 37 °C in a 5% CO_2_ atmosphere. The phenotypes of MSCs were analyzed by the flow cytometer, FACS CantoII, using a human MSC analysis kit (562245, BD Company, Franklin Lakes, NJ, USA) [38].

### 4.2. Processing of Allogeneic Bone

This study was approved by the Institutional Review Board of Taipei Veterans General Hospital, and informed consent was obtained from the donor following the tenets of the Declaration of Helsinki. Distal femurs were harvested from a patient who had undergone knee osteoarthritis replacement surgery (age 75). Examination for infection (hepatitis B virus, hepatitis C virus, human immunodeficiency virus, syphilis, and other infections) was conducted. After the removal of surrounding tissues, the distal femurs were cut into blocks (3 mm^3^) with a medical hand saw. The washing and γ-irradiation procedures were according to our previous study [21]. The sterilized blocks were stored at −80 °C.

### 4.3. HUVEC Culture

Human umbilical vein endothelial cells (HUVECs; ScienCell, Carlsbad, CA, USA) were cultured in endothelial cell media (ScienCell, Carlsbad, CA, USA) with 20% FBS and 1% of endothelial cell growth supplement (ECGS), 1% penicillin (100 μg/mL), and streptomycin (100 μg/mL) and incubated at 37 °C in a 5% CO_2_ atmosphere. HUVECs were passaged when the cells reached 90% confluence and used for subsequent experiments at passages 4–7.

### 4.4. Preparation of Conditioned Media

The MSC suspension (500 μL of 1 × 10^6^ cells in each well) was loaded onto an hDCB in a 48-well plate and grew in a-MEM media supplemented with 10% FBS. For the noninduced culture, MSCs were cultured in a-MEM media supplemented with 10% FBS for 14 days, which were the noninductive groups (nonOIM). For the osteo-induced culture, MSCs were cultured in osteo-inductive media (α-MEM containing 10% FBS and supplemented with 10^−8^ M dexamethasone (Sigma, St. Louis, MO, USA)) for 14 days after MSCs were seeded on hDCBs for 3 days, which were the osteo-inductive group (OIM). After being cultured in noninductive or osteo-inductive media for 14 days, the cell-seeded hDCBs were washed three times with phosphate-buffered saline (PBS). The media were replaced with 1 mL of serum-free a-MEM medium per hDCB. The next day, the conditioned media (CM) was centrifuged at 1000 rpm for 10 min at 4 °C, filtered with a 0.22 μm filter, and the concentrations of CMs were determined using the Bradford method. The collected CM was stored at −80 °C for subsequent experiments.

### 4.5. MTT Viability Assay

HUVECs were seeded at 2 × 10^5^ cells/mL in a 24-well plate. CMs (1 mg/mL) and endothelial cell growth media with 5% FBS (as positive controls) were added to triplicate wells and media were replaced every 3 days. Cell growth of HUVECs was evaluated at day 1, 3, 5, and 7 using a 3-(4,5-dimethylthiazolyl-2)-2,5-diphenyltetrazolium bromide (MTT, Sigma-Aldrich, St. Louis, MO, USA) assay. After several washes with PBS, cells were immersed in freshly prepared MTT reaction solution and incubated at 37 °C for 3 h in a CO_2_ incubator. The absorbance of each solution was detected at 570 nm using a Tecan Sunrise spectrophotometer (Tecan, Switzerland). Experiments were repeated three times (technical repeats).

### 4.6. Wound Healing Assay

The wound healing assay was performed based on a standard method [39] using HUVEC cells. In brief, a scratch was made vertically in each 100% of cell confluent well in a 24-well plate using a 200 mL disposable pipette tip, and then each well was washed 3 times with PBS. CMs or media with 5% FBS was added to the scratched wells. The wound areas were recorded using an inverted microscope with a 10 × objective, phase contrast filter, and digital camera at times 0 and 24 h, respectively. The area of the wound at times 0 h and 24 h was measured using ImageJ software. Experiments were repeated three times (technical repeats). The relative wound area was determined as the following:

% Wound area = (wound area (0 h) − wound area (24 h))/(wound area (0 h))

Relative wound area = % Wound area (CM-treated)/% Wound area (5% FBS)

### 4.7. Tube Formation Assay

A tube formation assay was based on an established protocol with modification [40]. Briefly, 200 mL of serum-starved (6 h) HUVEC cells were seeded onto a 96-well plate (2 × 10^4^ cells/well) precoated with 50 mL (10 mg/mL) growth-factor-reduced (GFR) Matrigel (Corning, NY, USA). After seeding, HUVEC cells were treated with 200 mL of ECGS media (as positive controls) or treated with MSC-CMs (1 mg/mL) and then incubated for 16 h at 37 °C in a 5% CO_2_ atmosphere. For the antibody neutralization experiment, the Abclonal IL-1b Rabbit mAb (Abclonal Inc., USA) was added to MSC-CMs at a ratio of 1:200, and then the HUVECs seeded on the GFR-Matrigel were treated with IL-1b antibody-containing MSC-CMs for 16 h at 37 °C in a 5% CO_2_ atmosphere. Images were taken under an inverted microscope with a digital camera. The captured image area (using a 10 × objective) was subjected to automatic image processing and data analyses by Fiji ImageJ (1.53t) with the angiogenesis analyzer plugin [41] to calculate the total number of meshes and nodes. For statistical analyses, CMs and ECGS were added to triplicate wells, and cells were treated with ECGS as positive controls. The data of CM-treated groups were normalized with ECGS groups. Data were expressed as the relative number of meshes and nodes. Experiments were repeated three times (technical repeats).

### 4.8. Real-Time Quantitative Polymerase Chain Reaction Analysis

The MSC suspension (500 μL of 1 × 10^6^ cells in each well) was loaded onto a human allogeneic bone block in a 48-well plate and grown in a-MEM media supplemented with 10% FBS. For noninductive culture, MSCs were cultured in a-MEM media supplemented with 10% FBS for 14 days. For osteo-inductive culture, MSCs were cultured in OIM for 14 days after MSCs seeded on hDCBs for 3 days. After being cultured in noninductive or osteo-inductive media for 14 days, the total RNA of MSCs was isolated using a TriRNA Pure Kit (Geneaid Biotech, New Taipei City, Taiwan), and cDNA was synthesized using an iScript^TM^ cDNA Synthesis Kit (Bio-Rad, Berkeley, CA, USA) according to the manufacturer’s instructions. A real-time quantitative polymerase chain reaction (RT-qPCR) was performed using a StepOnePlus real-time PCR System (Thermo) with the Smart Quant Green Master Mix with ROX (Protech technology, Taipei, Taiwan), under the following cycling conditions: 95 °C for 10 min, followed by 40 cycles at 95 °C for 10 s and 60 °C for 30 s. The cycle threshold for each gene of interest was normalized against that of the housekeeping gene (*GAPDH*), and relative gene expression levels were determined using the 2^−ΔΔCt^ method. Experiments were repeated three times (technical repeats). The primer pairs of genes analyzed are listed in Table 1.

### 4.9. Statistical Analysis

GraphPad Prism v7.0 (GraphPad Software, San Diego, CA, USA) was used for statistical analysis. The one-way ANOVA with Turkey’s post hoc test was used to examine the differences between the experimental groups. Significance was set at *p* < 0.05. Heatmaps and principal component analysis were plotted using MeV (Multi Experiment Viewer). The analysis of differentially expressed genes and the hierarchical clustering among MSCs were performed using ANOVA (*p* < 0.05) with average linkage, permutations (1000), and Pearson correlation. To compare the differentially expressed genes between nonOIM and OIM groups and between BMMSCs and other MSCs, the *t*-test was used. Color scales are log2^−ΔΔCt^, where red indicates low expression levels and blue indicates high expression levels. For IL-1b neutralization experiments, the *t*-test was employed to compare untreated and treated samples (*p* < 0.05).

## 5. Conclusions

In this study, we sought to investigate the angiogenic potential of MSC secretomes based on the tissue of origin and the state of differentiation to see if they had underlying differences. Our data show that the secretomes of MSCs seeded on human decellularized allogeneic bone do induce endothelial cell proliferation, migration, and angiogenesis. Moreover, one of the angiogenic molecules, IL-1b, plays an important role in angiogenesis. We also demonstrate that the expression of angiogenic genes is with tissue-dependent profile, but the angiogenic potential of MSC secretomes is independent of the state of differentiation. The secretome of MSCs has been emerging as a potential cell-free treatment in tissue regeneration. In addition, the regulation of IL-1b expression in injured tissue might be a promising treatment to induce angiogenesis and enhance tissue repair in future clinical practice.

## Figures and Tables

**Table 1:** Primer pairs for real-time quantitative polymerase chain reaction.

Gene Name	Forward Primer (5′ to 3′)	Reverse Primer (5′ to 3′)	Accession No.	Product Size (bp)
ANG	ACTGGAACCCATCTCCAGGAACA	CAACACAGGCTCCCAGGAGGAA	NM_001097577	100
ANGPT1	TCAGTGGCTGCAAAAACTTGAGA	CAGCATGGTAGCCGTGTGGT	NM_001146	103
ANGPT2	GCTAAGGACCCCACTGTTGCTA	TCCATGTCACAGTAGGCCTTGAT	NM_001147	146
ARTN	GCTGTTTGAGCTTCGGGGGA	TGTTCCCCCACCCTCCTGTT	NM_057091	104
CCL13	ACAGCAGCTTTCAACCCCCA	TGAAGCAGCAAGTAGATGGGACG	NM_005408	70
CCL3	GGTGTCATCTTCCTAACCAAGCGAA	CTCAGGCACTCAGCTCCAGGTC	NM_002983	100
CCL7	AGGCTGGAGAGCTACAGAAGGAC	CTGTGTGGGGTCAGCACAGAT	NM_006273	102
COL18A1	CGACTTCCAGCCGGTGCTC	GACAGGAAGGCGCGGAAGG	NM_001379500	141
CSF2	CGGAAACTTCCTGTGCAACCC	CCTCATCTGGCCGGTCTCACT	NM_000758	126
CSF3	CTGGACAGTGCAGGAAGCCAC	TAGGTGGCACACAGCTTCTCCT	NM_172219	135
EDN1	GCCAAAAAGACAAGAAGTGCTGG	TCCATAATGTCTTCAGCCCTGAGTT	NM_001955	70
EGF	AGGAGAACATCTCTCAACCACGAG	AGGCCTTGGAGGGAAGAACTTT	NM_001963	112
ENG	AGCCCCACAAGTCTTGCAGAA	CACGCAGCCCTTCGAGACCT	NM_001114753	105
FGF2	CGGGTGCAGTGGCTCATGCCTATA	CGGGGTTTCACCAGGTTGGTCTTG	NM_002006	100
FGFR1	GCATGGTGGGGTCGGTCATC	CTGGAGTCAGCAGACACTGTTACC	NM_023110	136
FLT1	TGACCCACATTGGCCACCATC	GTGTAGTGCTGCATCCTTGTTGA	NM_002019	167
HGF	CAGCATCATCGAGGGAAGGTGACTC	CCCCTCACATGGTCCTGATCCAA	NM_000601	84
HIF1A	GACATCGCGGGGACCGATTC	CGCCGAGATCTGGCTGCAT	NM_001530	112
IGF1	AGCTGGTGGATGCTCTTCAGTTCG	CACTCATCCACGATGCCTGTCTGA	NM_001111283	115
IGFBP1	CAACCTCTGCACGCCCTCAC	TCTCCGTGCTCTCTGGGCTT	NM_000596	112
IGFBP2	CTCCCTGCACATCCCCAACTG	CTTCCCGGTGTTGGGGTTCA	NM_000597	115
IGFBP3	ACCACCAAGGGGAAGGAGGA	AGCTGCTGGTCATGTCCTTGG	NM_000598	130
IL-1A	TGTGACTGCCCAAGATGAAGACCA	TGCCGTGAGTTTCCCAGAAGAAGAG	NM_000575	113
IL-1B	GAAGTACCTGAGCTCGCCAGT	GCCTGAAGCCCTTGCTGTAGT	NM_000576	181
IL-4	GAGAAGGACACTCGCTGCCTG	GAGGTTCCTGTCGAGCCGTT	NM_000589	93
IL-8	GACCACACTGCGCCAACACAGAA	CCACAACCCTCTGCACCCAGTTT	NM_000584	96
KDR	CAAGTGGCTAAGGGCATGGA	ATTTCAAAGGGAGGCGAGCA	NM_002253	181
LEP	TAGGAATCGCAGCGCCAGC	TGTGTGAAATGTCATTGATCCTGGT	NM_000230	198
MMP8	CCAGCAACTACTCACTCCCTCAAG	CAGGGTTTGGGTGTGCTTGGT	NM_002424	106
PDGFA	ATCGGGAAGAGGACACGGGAA	ATCTGGTTGGCTGCTTTAGGTGG	NM_002607	89
PDGFB	ACCACCTGGCATGCAAGTGTGAGAC	TCCGAATGGTCACCCGAGTTTGG	NM_002608	114
PDGFC	CTGAACCAGGGTTCTGCATCCACT	AGGGGGTAGCACTGAAGGACTCACA	NM_016205	80
PDGFD	GGAAGATTTCCAACCCGCAGCA	GTCCAGAGCATCCGCAATCAGAGT	NM_025208	124
PLAU	GGACCCCTCGTCTGTTCCCT	GGTGTGACTGCGGATCCAGG	NM_002658	138
PLG	GCTGACCGGACCGAATGTTT	GAGTTCGGTGGATTGGACTCTTCC	NM_000301	153
PRL	GGCTTCTAGAGGGCATGGAGC	CGTAGGCAGTGGAGCAGGTT	NM_000948	154
PTX3	GGCAGACGCGAGCCGA	AAGCCTCATTGGTCTCACTGGATG	NM_002852	143
SERPINB5	TGGAGGCCACGTTCTGTATGG	GGGTAGTAGGATGAACATGCTGAG	NM_002639	101
SERPINE1	GCCCATGATGGCTCAGACCAA	CTGAGGGTGTCCCCGTGGTA	NM_000602	105
SERPINF1	AGGCGAAGTCACCAAGTCCC	TCAAAGCCAGCCCGGTGTTC	NM_002615	123
TEK	TGCCACCATCACTCAGTATCAGCTC	TCCGCTGGTTGCTTGAGATTCTG	NM_000459	147
THBS1	TGCTGGTGGTAGACTAGGGTTGTT	ATCCTGGGGGTTTTCTCAAGCC	NM_003246	173
THBS2	GGGCGGCTGGGTCTATTTGT	GCACAGGGCATTGCCGGA	NM_003247	113
THPO	TGGGTCCTGGAGCCCTTCTC	GGAGGCGGCTTAGGCTCTTG	NM_000460	101
TIE1	GTACGAGCTGATGCGTCAGTGCT	GCCCGCGTAAGTGAAGTTCTCA	NM_005424	145
VEGFA	CCATGCCAAGTGGTCCCAGG	GATGGCAGTAGCTGCGCTGA	NM_001025366	104
VWF	CAATGAGTTCCAACTGCAGCTCAGC	TGCCATCCCTCAGCATGAAGTCA	NM_000552	110

## Data Availability

The data presented in this study are available on request from the corresponding author.
